# Microarray Genotyping Identifies New Loci Associated with Dementia in Parkinson’s Disease

**DOI:** 10.3390/genes12121975

**Published:** 2021-12-10

**Authors:** Sungyang Jo, Kye Won Park, Yun Su Hwang, Seung Hyun Lee, Ho-Sung Ryu, Sun Ju Chung

**Affiliations:** 1 Department of Neurology, Asan Medical Center, University of Ulsan College of Medicine, Seoul 05505, Korea; sungyangjo@gmail.com (S.J.); ghkddbstn1@naver.com (Y.S.H.); doors327@naver.com (S.H.L.); 2 Department of Neurology, Uijeongbu Eulji Medical Center, Eulji University School of Medicine, Uijeongbu-si 11759, Gyeonggi-do, Korea; karabach88@gmail.com; 3 Department of Neurology, Kyungpook National University Hospital, Daegu 41944, Korea; ryuhosung138@gmail.com

**Keywords:** genome-wide association study, Parkinson’s disease, dementia, cognition

## Abstract

Dementia is one of the most disabling nonmotor symptoms of Parkinson’s disease (PD). However, the risk factors contributing to its development remain unclear. To investigate genetic variants associated with dementia in PD, we performed microarray genotyping based on a customized platform utilizing variants identified in previous genetic studies. Microarray genotyping was performed in 313 PD patients with dementia, 321 PD patients without dementia, and 635 healthy controls. The primary analysis was performed using a multiple logistic regression model adjusted for age and sex. *SNCA* single nucleotide polymorphism (SNP) rs11931074 was determined to be most significantly associated with PD (odds ratio = 0.66, 95% confidence interval = 0.56–0.78, *p* = 7.75 × 10^−7^). In the analysis performed for patients with PD only, *MUL1* SNP rs3738128 (odds ratio = 2.52, 95% confidence interval = 1.68–3.79, *p* = 8.75 × 10^−6^) was found to be most significantly associated with dementia in PD. SNPs in *ZHX2* and *ERP29* were also associated with dementia in PD. This microarray genomic study identified new loci of *MUL1* associated with dementia in PD, suggesting an essential role of mitochondrial dysfunction in the development of dementia in patients with PD.

## 1. Introduction

Parkinson’s disease (PD) is the second most prevalent neurodegenerative disease globally, affecting more than six million people worldwide [1]. The diagnosis of PD is based on specific motor symptoms, including bradykinesia, rigidity, tremor, or gait disturbance [2]. However, patients with PD suffer from various nonmotor symptoms, such as fatigue, pain, sleep disturbance, dementia, depression, anxiety, and autonomic dysfunction [3]. Dementia is one of the disabling nonmotor symptoms that substantially impairs the quality of life of patients with PD, increasing caregiver burden and economic costs [4]. The prevalence of dementia is high, with up to 75% of patients with PD developing dementia within 10 years from diagnosis [5,6]. However, the determining factors involved in the development of dementia in patients with PD are still unclear.

Genome-wide association studies (GWAS) have widened our understanding of the genetics of PD and have identified more than 90 genetic loci that are associated with the development of PD [7,8,9,10,11,12,13]. However, a majority of the previously conducted GWAS have focused on the susceptibility of PD, and GWAS specifically investigating motor or nonmotor presentations—including dementia—of PD have been limited. In a recent GWAS, we reported that *RYR2* and other genetic loci are associated with cognitive impairment in PD; however, the assessment of cognitive function was based only on Mini-Mental Status Examination (MMSE) and the Montreal Cognitive Assessment (MoCA) scores [14]. Many recent GWAS have reported genomic variants, including *GBA*, *APOE*, *SNCA*, and *CNTN1* that are associated with dementia with Lewy bodies (DLB) [15,16,17,18]. Although Parkinson’s disease dementia (PDD) and DLB share clinical, neurochemical, and morphological features, no consensus has been established yet with respect to the consideration of the two extremes on the one continuous spectrum of Lewy body disease [19]. Interestingly, in a large multinational cohort of patients with PD, PDD, and DLB, parkinsonism and dementia showed two distinct association profiles with the 3′ or 5′ regions of the *SNCA* gene, suggesting that PD, PDD, and DLB have distinct genetic etiologies. Therefore, further studies undertaking genome-wide investigations are necessary to identify distinct genetic variants associated with the development of dementia in patients with PD, independent of DLB.

In this study, we employed a novel customized microarray platform to comprehensively investigate the genetic variants associated with dementia in patients with PD. 

## 2. Materials and Methods

### 2.1. Study Population

We prospectively enrolled patients with PDD, patients with PD without dementia (PD-ND), and healthy controls at Asan Medical Center, Seoul, Korea. All participants were ethnic Koreans. The diagnosis of PD was based on the UK Brain Bank criteria [2] and the diagnosis of PDD was based on the criteria proposed by the Movement Disorder Society Task Force [20]. Healthy controls were recruited from among the spouses of the patients, and the inclusion criterion included the absence of neurological diseases including PD or dementia. Blood samples were collected from all participants for genetic tests, and patient information including that related to age, sex, and educational qualification (number of years of education) was collected at the time of sampling. Mini-Mental Status Examination (MMSE) was performed for the screening of cognitive function. For patients with PD, age at disease onset, age at diagnosis of dementia if applicable, age at latest follow-up, and the latest MMSE scores were obtained. 

### 2.2. Development of Microarray Genotyping Platform

We designed a microarray genotyping platform that contained genetic variants with biological plausibility for PDD, suggested by our previous GWAS or other previous genetic studies. The platform included: (1) Genetic variants that showed a high level of association (*p*-value < 10^−4^) with PD in our previous GWAS performed using ethnicity-specific Korean Chip (K-CHIP). K-CHIP was designed by the Center for Genome Science, Korea National Institute of Health (4845–301, 3000–3031) (www.cdc.go.kr) [14,21]. K-CHIP consists of an imputation GWAS grid (505,000 Asian-based grid with minor allele frequency (MAF) > 5% in Asians); exome content (84,000 Korean-based grid with MAF > 5% in Koreans, 149,000 coding single-nucleotide polymorphisms, and insertions and deletions determined based on data derived from 2000 whole-exome sequences and 400 whole-genome sequences with MAF > 0.1%); new exome/loss of function contents (44,000 variants); expression quantitative trait loci (17,000 variants); genes associated with absorption, distribution, metabolism, and excretion; and other miscellaneous variants. (2) Genetic variants that showed significant association with PD in previous GWAS [7,8,9,10,11,12,13]. (3) Genetic mutations that were reported to be a cause of monogenic familial PD with Mendelian inheritance (https://www.omim.org/). (4) Genetic variants that showed significant association with DLB in previous GWAS [15,16,22]. (5) Genetic variants that showed significant association with Alzheimer’s disease in previous GWAS [23,24,25,26]. (6) Genetic variants associated with neuroinflammation in previous GWAS [11,27,28]. 

Annotation of the variants was performed using the nspEff tool to confirm the distribution of the gene effect [29]. From a total of 219,065 variants, we excluded 109,804 “novel—not recommended and neutral” markers for the score data, because the performance or efficacy of genotyping might be low (Table S1). The final selection was performed by excluding duplicate markers, markers not included in the 1000 genome project phase 3 data, markers with a minor allele frequency of zero in East Asian GWAS data, and proxy single nucleotide polymorphisms (SNPs) (tagging *r*^2^ > 0.8) (Table S2). The final candidate markers consisted of 74,224 markers (Table S3). 

### 2.3. Sample Quality Control

Samples with a low call rate and high heterozygosity were excluded. Samples that deviated from the whole sample were excluded from the analysis by an assessment performed using multidimensional scaling. We also excluded excessive singleton, samples with gender discrepancies, and cryptic first-degree relatives using the PLINK program (version 1.90, NIH–NIDDK Laboratory of Biological Modeling, Bethesda, MD, USA).

### 2.4. SNP Quality Control

We performed an SNPolisher analysis to exclude low-quality SNPs. SNPs with call rates over 95% in both cases and controls were included. SNPs with *p*-value > 10^−4^ in a Hardy–Weinberg equilibrium test were excluded. We excluded SNPs with minor allele frequency < 1% in both cases and controls. We performed cluster quality control for every SNP with *p* < 0.001 using linkage disequilibrium within 150 kilobases through visual inspection.

### 2.5. Statistical Analysis

We compared the demographics and clinical characteristics of patients with PDD, those with PD-ND, and healthy controls using Kruskal-Wallis tests for continuous variables, which did not meet the assumption of the homogeneity of variance, as well as with chi-squared test for categorical variables. Post hoc analysis was performed using Dunnett’s post hoc tests and Bonferroni correction. 

The association between the genetic variants and PD or PDD was analyzed using a multiple logistic regression model after adjusting for age, sex, and education years. For each genetic variant, we calculated the odds ratios (OR), 95% confidence interval (CI), and two-tailed *p*-value. Bonferroni correction was applied to adjust for multiple comparisons. Manhattan plots and quantile–quantile plots (Q-Q plots) were constructed for *p*-values of all genotyped variants that passed quality control. 

Statistical analysis was performed using R (version 3.1.2, Free Software Foundation, Inc., Boston, MA, USA), the PLINK program (version 1.90, NIH–NIDDK Laboratory of Biological Modeling, Bethesda, MD, USA), Haploview (version 4.2, Daly Lab at the Broad Institute, Cambridge, MA, USA), and LocusZoom (version 1.4, University of Michigan, Department of Biostatistics, Center for Statistical Genetics, Ann Arbor, MI, USA).

## 3. Results

### 3.1. Clinical Characteristics

We enrolled 318 patients with PDD, 326 patients with PD-ND, and 648 healthy controls. After quality control assessment, 5 patients with PDD, 5 patients with PD-ND, and 13 healthy controls were excluded. The final study population included 313 patients with PDD, 321 patients with PD-ND, and 635 healthy controls. The ages noted at the latest follow-up for patients with PD or those noted at study enrollment for healthy controls were significantly different among the three groups (median 76.0 vs. 75.0 vs. 68.0, *p* < 0.001) (Table 1). In the post hoc analysis, ages noted at the latest follow-up were significantly lower among healthy controls than those among patients with PDD or PD-ND (all *p* < 0.001). The ages at disease onset and the disease durations were not significantly different between patients with PDD and PD-ND. The median disease duration was 12.0 years for both PDD and PD-ND groups. The percentage of females was significantly higher in the PDD group compared to that in the healthy controls (57.2% vs. 44.9%, *p* = 0.0007 in post hoc analysis). The number of education years (total years of academic education) was significantly lower in the PDD group than that in the PD-ND or healthy control group (both *p* < 0.001 in post hoc analysis). 

### 3.2. Genetic Association with Susceptibility to PD

**Figure 1:**
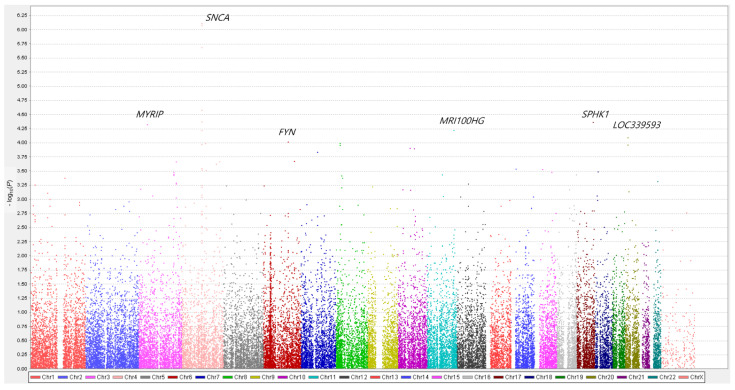
Manhattan plots for Parkinson’s disease (PD). The genes nearest to the top 10 significant variants are labeled. The *x*-axis represents the base pair position of the variants from chromosome 1 to chromosome 22. The *SNCA* loci showed a statistically significant association with PD after Bonferroni correction. *SNCA* SNP rs11931074 was most significantly associated with PD (OR = 0.66, 95% CI = 0.56–0.78, *p* = 7.75 × 10^–7^). The *SPHK1* and *FYN* loci were also associated with PD.

The 41,534 genetic variants that passed quality control were genotyped and analyzed. Multiple logistic regression with additive coding schemes was performed to compare genetic variants between patients with PD (both, patients with PDD and those with PD-ND) and healthy controls after adjusting for age and sex. Q-Q plots were generated for the diagnosis of patients with PD in comparison with healthy controls (Figure S1). The Manhattan plot is depicted in Figure 1. Among the top 10 genetic variants associated with PD, five SNPs were observed in the loci of *SNCA* (rs11931074, rs12642514, rs75876872, rs80184884, and rs75231811) (Table 2), and two *SNCA* SNPs (rs11931074 and rs12642514) showed statistical significance after Bonferroni correction (Figure 1). Among the *SNCA* SNPs, SNP rs11931074 was most significantly associated with PD (OR = 0.66, 95% CI = 0.56–0.78, *p* = 7.75 × 10^–7^). *SPHK1* SNP rs2247856 (OR = 0.65, 95% CI = 0.53–0.80, *p* = 4.35 × 10^–5^) and *FYN* SNP rs7772036 (OR = 0.72, 95% CI = 0.61–0.85, *p* = 9.74 × 10^–5^) were also associated with PD.

### 3.3. Genetic Association with Dementia in PD

**Figure 2:**
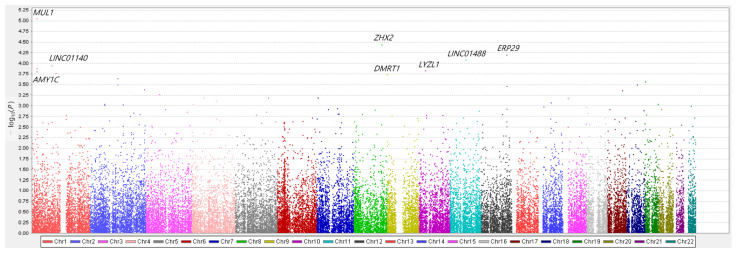
Manhattan plots for dementia in Parkinson’s disease (PD). The genes nearest to the top 10 significant variants are labeled. The *x*-axis represents the base pair position of the variants from chromosome 1 to chromosome 22. The *MUL1* loci was most significantly associated with dementia in PD. *MUL1* SNP rs3738128 (OR = 2.52, 95% CI = 1.68–3.79, *p* = 8.75 × 10^–6^) was most significantly associated with dementia in PD. The *ZHX2* and *ERP29* loci were also associated with dementia in PD.

**Figure 3:**
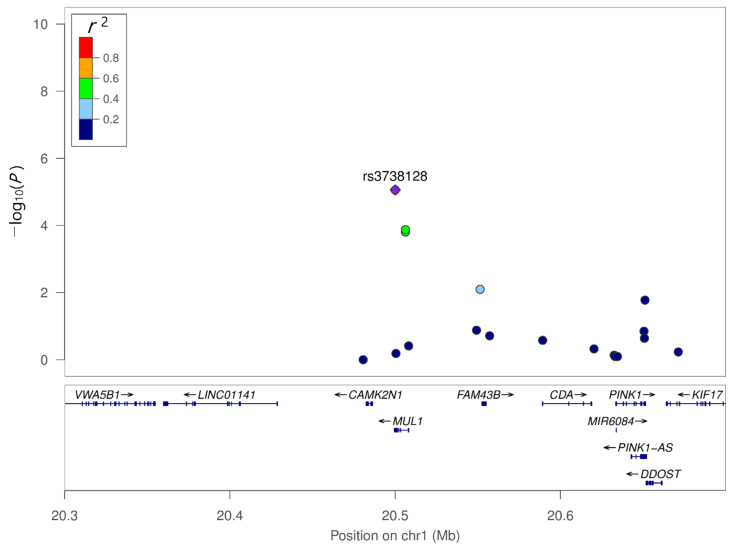
Regional association plot of the genetic variants of *MUL1*. *MUL1* SNP rs12566937 showed moderate linkage disequilibrium with *MUL1* SNP rs3738128.

We compared genetic variants between PDD and PD-ND using multiple logistic regression with additive coding schemes after adjusting for age, sex, and education years. Q–Q plots were generated for the diagnosis of PDD compared with PD-ND (Figure S2). The respective Manhattan plot is depicted in Figure 2. Among the top 10 SNPs associated with PDD, two SNPs were observed in the loci of *MUL1* (rs3738128 and rs12566937) (Table 3). *MUL1* SNP rs3738128 (OR = 2.52, 95% CI = 1.68–3.79, *p* = 8.75 × 10^6^) was most significantly associated with dementia in PD. In linkage analysis, *MUL1* SNP rs12566937 showed moderate linkage disequilibrium with *MUL1* SNP rs3738128, which was associated with the lowest *p*-value (Figure 3). SNPs in *ZHX2* (OR = 0.56 95% CI = 0.43–0.74, *p* = 3.65 × 10^–5^) and *ERP29* (OR = 3.05, 95% CI = 1.77–5.27, *p* = 6.41 × 10^–5^) were also associated with dementia in PD. However, following Bonferroni correction, none of the SNPs showed statistical significance.

## 4. Discussion

In this study, we identified genetic variants that were significantly associated with dementia in patients with PD with a median disease duration of over 12 years. The *MUL1* SNP rs3738128 showed the most significant association with dementia in PD. *ZHX2* and *ERP29* also showed correlations with dementia in PD. The *SNCA* locus showed the most significant association with susceptibility to PD, consistent with the results of previous GWAS [7,8,9,10,11,12].

There were few studies investigating the role of *MUL1* in the development of dementia in PD, and one case-control study conducted in China showed that *MUL1* SNP rs529974 was correlated with the development of PD [30]. *MUL1* encodes mitochondrial ubiquitin ligase 1, a mitochondrial E3 protein ligase that regulates mitofusin. The mitochondria are involved in cellular energy production and cell survival, playing an important role in the neurodegenerative process in PD [31]. Mitochondrial genes such as *parkin, PINK1, DJ-1, LRRK2, ATP13A2*, and *VPS35* are associated with PD [32]. An experimental study showed that *MUL1* suppressed the mitochondrial phenotype in *PINK1/parkin* mutant dopaminergic neuron, and the knockdown of *MUL1* in *parkin* knockout mouse cortical neurons augmented mitochondrial damage [33]. Therefore, mutants with *MUL1* and *parkin* mutations are employed in the development of animal models of PD [34]. *MUL1* overexpression has been shown to reduce the degeneration of dopaminergic neurons and enhance motor activity in neurons of flies fed with rotenone [35]. *MUL1* dysfunction renders dopaminergic neurons susceptible to mitochondrial damage. The loss of *MUL1* function may be more prominent when other mitochondrial dysfunctions exist as well, as a result of genetic variants or environmental toxins. The lack of correlation of *MUL1* with PD in this study may be explained by the adjunctive role of *MUL1* in mitochondrial function.

Considering that the *MUL1* pathway regulates mitochondrial damage in both dopaminergic and cortical neurons [33,36], defects in the *MUL1* pathway might affect the cognitive decline in PD. However, little is known about the association between *MUL1* and cognitive decline in PD or other neurodegenerative diseases that cause dementia. Mitochondrial dysfunction induces energy deficiency, intracellular calcium imbalance, and oxidative stress, leading to synaptic dysfunction and neuronal cell loss [37]. This mechanism explains how mitochondrial dysfunction mediates cognitive decline in neurodegenerative diseases, such as Alzheimer’s disease. Mitochondrial dysfunction is also prominent among patients with PD [38]. When *MUL1* is downregulated, cortical neurons, as well as dopaminergic neurons, might become more susceptible to damage due to mitochondrial dysfunction, leading to the progression of cortical neuronal loss, synaptic dysfunction, and cognitive decline. In addition, recent studies have revealed that amyloid-beta and p-tau interact with mitochondrial proteins, resulting in increased mitochondrial fragmentation and reduced mitochondrial fusion in Alzheimer’s disease [39]. Similarly, pathogenic alpha-synuclein and amyloid-beta found in the brains of patients with PDD [40,41] might interact with *MUL1*, leading to mitochondrial dysfunction. The significant association of *MUL1* with dementia in PD suggests the biological plausibility of the involvement of mitochondrial dysfunction in the development of dementia in PD. Further studies are needed to elucidate the exact pathogenic mechanisms underlying the involvement of *MUL1* in the development of dementia in PD.

Other genetic variants associated with dementia in PD were located in the loci of *ZHX2* and *ERP29*. Few clinical studies have investigated the role of *ZHX2* and *ERP29* in PD or dementia. The gene *ZHX2* encodes zinc-finger and homeodomain protein 2 (ZHX2) that regulates transcription and neuronal differentiation [42]. Genetic variants of *ZHX2* were found in two affected members of familial corticobasal degeneration, mutations of which were predicted to impair protein function [43]. Both corticobasal degeneration and PD are neurodegenerative diseases characterized by damage to cortical neurons and cognitive decline. Since *ZHX2* is also associated with cortical neuronogenesis [42], it may be associated with the progression to dementia. *ERP29* gene encodes a 29 kDa endoplasmic reticulum protein (ERp29), which is ubiquitously expressed in cells and regulates protein transport between the endoplasmic reticulum and Golgi apparatus [44]. ERp29 is involved in protein misfolding and mistrafficking [44,45], which are potent pathogenic features of PD and Alzheimer’s disease [46]. Given that endoplasmic reticulum stress is related to Lewy body dementia [47], it is possible that ERp29 mutation also induces cortical neuronal damage and is linked to the progression of dementia in patients with PD. 

In our study, the *SNCA* SNP rs11931074 was most significantly associated with susceptibility to PD, which is consistent with previous results [7,9,10,11,12,48]. Mutations in the *SNCA* gene were first found in familial PD with autosomal dominant inheritance [49,50], and several SNPs across the *SNCA* locus were also linked to the increased risk for sporadic PD in multiple GWAS [7,8,9,10,11,12]. The *SNCA* gene encodes alpha-synuclein, which is the main component of Lewy bodies, the pathologic hallmark of PD. Interestingly, *SNCA* SNP rs11931074, which showed the most significant association with PD in this study also has a distinct relationship with PD based on race [48]. The presence of *SNCA* SNP rs11931074 increases the risk of PD, as demonstrated by the allele model, homozygote model, and recessive model developed for the Asian population, while the association was found to be true only in an allele model developed for the Caucasian population. These results support the quality of PD samples used in this study and might emphasize the role of *SNCA* SNP rs11931074 in the development of PD in the Asian population.

We found that *SPHK1* and *FYN* SNPs were associated with PD. *SPHK1* gene encodes sphingosine kinase 1 protein, which phosphorylates sphingosine into sphingosine-1-phosphate (S1P). S1P synthesized by *SPHK1* exerts mitogenic and anti-apoptotic effects in an autocrine or paracrine manner [51]. The expression of sphingosine kinase 1 was downregulated in experimental models of PD, and inhibition of sphingosine kinase 1 decreases cell viability and enhances the production of reactive oxygen species [52]. *FYN* gene encodes the Fyc protein, which is a tyrosine phosphotransferase enzyme belonging to the Src family of nonreceptor tyrosine kinases. Fyc has been suggested to regulate alpha-synuclein phosphorylation, oxidative stress-induced dopaminergic neuronal death, and enhancement of neuroinflammation [53]. Therefore, both sphingosine kinase 1 protein and Fyc were suggested as potential therapeutic targets for PD [51,53], and our data support the protective effects of *SPHK1* and *FYN* in PD.

The strength of this study is that we used clinical diagnosis of dementia based on the long-term follow-up of patients with PD. The prevalence of dementia in patients with PD is 17% at 5 years after diagnosis and 46−75% at 10 years after diagnosis [6,54]. Therefore, including PD patients with a short follow-up duration would misclassify them as having PD without dementia. A previous GWAS investigating the cognitive decline in PD included patients whose median follow-up duration was 4 years [55], and another GWAS assessed cognition using cross-sectional MMSE scores or MoCA scores [14]. 

This study has a few limitations. First, the sample size was relatively small, which may explain why genetic variants associated with dementia in patients with PD did not remain statistically significant after stringent Bonferroni correction. Second, the biological functions of the genetic variants were not validated. However, the experimental studies on *SPHK1*, *FYN*, *MUL1*, *ZHX2*, and *ERP29* genes, as discussed above, might support the biological plausibility of the involvement of these genes in PD. Therefore, future functional studies are required to confirm our results.

## 5. Conclusions

This microarray genomic study identified the new loci of *MUL1* associated with dementia in PD, suggesting an essential role of mitochondrial dysfunction in the development of this nonmotor symptom of PD.

## Figures and Tables

**Table 1:** Baseline clinical characteristics of the study subjects.

Characteristics	PD Dementia (*N* = 313)	PD without Dementia (*N* = 321)	Controls (*N* = 635)	*p*-Value
Age at onset, years	64.0 (57.0−68.0)	63.0 (57.0−68.0)	-	0.449
Age at latest follow-up, years	76.0 (72.0–81.0)	75.0 (72.0−80.0)	68.0 (64.0−72.0) ^a,b^	<0.001
Disease duration, years	12.0 (9.0−17.0)	12.0 (9.0−16.0)	-	0.896
Female, *N* (%)	179 (57.2%)	166 (51.7%)	285 (44.9%) ^a^	0.001
Education, years	6.0 (2.0−12.0)	12.0 (6.0−16.0) ^c^	12.0 (9.0−16.0) ^a^	<0.001
Latest MMSE	17.0(13.0−20.0)	27.0 (26.0−29.0) ^c^	28.0 (26.0−29.0) ^a^	<0.001
Age at dementia, years	73.0 (69.0−78.0)	-	-	

PD, Parkinson’s disease; MMSE, Mini-Mental Status Examination. ^a^ Significant difference compared with PD dementia using Dunn’s post hoc test. ^b^ Significant difference compared with PD without dementia using Dunn’s post hoc test. ^c^ Significant difference compared with healthy controls using Dunn’s post hoc test.

**Table 2:** Top 10 genetic variants associated with Parkinson’s disease in the order of statistical significance.

Gene	SNP	Chr	Position	Region Relative to the Gene	Allele (Minor/Major)	Minor Allele Frequency (Case/Control)	OR (95% CI)	*p*-Value
*SNCA, GPRIN3*	rs11931074	4	89718364	intron, downstream, upstream	G/C	0.37/0.46	0.66 (0.56, 0.78)	7.75 × 10^–7^
*SNCA, GPRIN3*	rs12642514	4	89708246	intron, downstream, upstream	A/C	0.36/0.46	0.66 (0.58, 0.79)	2.08 × 10^–6^
*SNCA*	rs356191	4	89766969	Intron	A/G	0.06/0.10	0.52 (0.38, 0.70)	2.64 × 10^–5^
*SNCA, GPRIN3*	rs80184884	4	89705068	intron, downstream, upstream	G/A	0.06/0.10	0.52 (0.38 0.71)	4.24 × 10^–5^
*SPHK1*	rs2247856	17	76385474	missense, UTR-5, exon	A/G	0.16/0.22	0.65 (0.53, 0.80)	4.35 × 10^–5^
*MYRIP*	rs6599077	3	40055127	Intron	A/G	0.43/0.35	1.42(1.20, 1.68)	4.81 × 10^–5^
*MRI100HG*	rs577924	11	122264399	Intron	C/T	0.43/0.35	1.41 (1.19, 1.67)	6.05 × 10^–5^
*SNCA, GPRIN3*	rs75876872	4	89705795	intron, downstream, upstream	G/A	0.05/0.08	0.49 (0.35, 0.69)	6.07 × 10^–5^
*LOC339593*	rs1473702	20	11253884	intron, downstream	C/T	0.51/0.44	1.38 (1.18, 1.62)	8.05 × 10^–5^
*FYN*	rs7772036	6	111739596	Intron	G/A	0.32/0.39	0.72 (0.61, 0.85)	9.74 × 10^–5^

Chr, chromosome; OR, odds ratio; CI, confidence interval.

**Table 3:** Top 10 genetic variants associated with dementia in Parkinson’s disease in the order of statistical significance.

Gene	SNP	Chr	Position	Region Relative to the Gene	Allele (Minor/Major)	Minor Allele Frequency (Case/Control)	OR (95% CI)	*p*-Value
*MUL1*	rs3738128	1	20499992	UTR-3	G/C	0.07/0.11	2.52 (1.68, 3.79)	8.75 × 10^–6^
*ZHX2*	rs11779459	8	122968311	Intron	T/C	0.34/0.29	0.56 (0.43, 0.74)	3.65 × 10^–5^
*ERP29, NAA25*	rs4767293	12	112025492	downstream	A/G	0.04/0.06	3.05 (1.77, 5.27)	6.41 × 10^–5^
*LINC01488*	rs7395791	11	69448148	upstream, downstream	A/G	0.56/0.50	0.61 (0.47, 0.78)	8.44 × 10^–5^
*LINC01140*	rs7553864	1	87147675	Intron	T/C	0.14/0.19	1.88 (1.37, 2.6)	1.15 × 10^–4^
*MUL1*	rs12566937	1	20506181	Intron	G/T	0.13/0.17	1.91 (1.37, 2.67)	1.33 × 10^–4^
*LYZL1, C10orf126*	rs1889714	10	29099710	upstream, downstream	A/G	0.12/0.09	0.43 (0.28, 0.66)	1.47 × 10^–4^
*AMY1C, LOC101928476, LOC100129138*	rs12026039	1	104028469	downstream, upstream	G/A	0.51/0.47	0.61 (0.47, 0.79)	1.74 × 10^–4^
*DMRT1, KANK1*	rs912062	9	841152	upstream, downstream	C/A	0.17/0.22	1.76 (1.31, 2.37)	1.82 × 10^–4^
*GLI2, LINC01101*	rs11688682	2	120590036	Upstream	C/G	0.08/0.04	2.62 (1.57, 4.37)	2.30 × 10^–4^

SNP, single-nucleotide polymorphism; Chr, chromosome; OR, odds ratio; CI, confidence interval.

## Data Availability

The data presented in this study are available on request from the corresponding author. The data are not publicly available due to the privacy of the study population.

## Supplementary Materials

The following are available online at https://www.mdpi.com/article/10.3390/genes12121975/s1, Figure S1: Quantile–quantile plot for Parkinson’s disease, Figure S2: Quantile–quantile plot for dementia in Parkinson’s disease, Table S1: Characteristics of the markers used in the microarray, Table S2: Staged verification of the markers, Table S3: Additional selection according to selection priority.
