# Supplementary material for: Microarray Genotyping Identifies New Loci Associated with Dementia in Parkinson’s Disease
Source: Genes (Basel). 2021 Dec 10;12(12):1975. doi: 10.3390/genes12121975 (PMC8701809; doi:10.3390/genes12121975)
Supplement: Supplementary file 1 [file genes-12-01975-s001.zip › genes-1452527-supplementary - final-done.pdf]

genes

MDPI

# Supplementary Materials

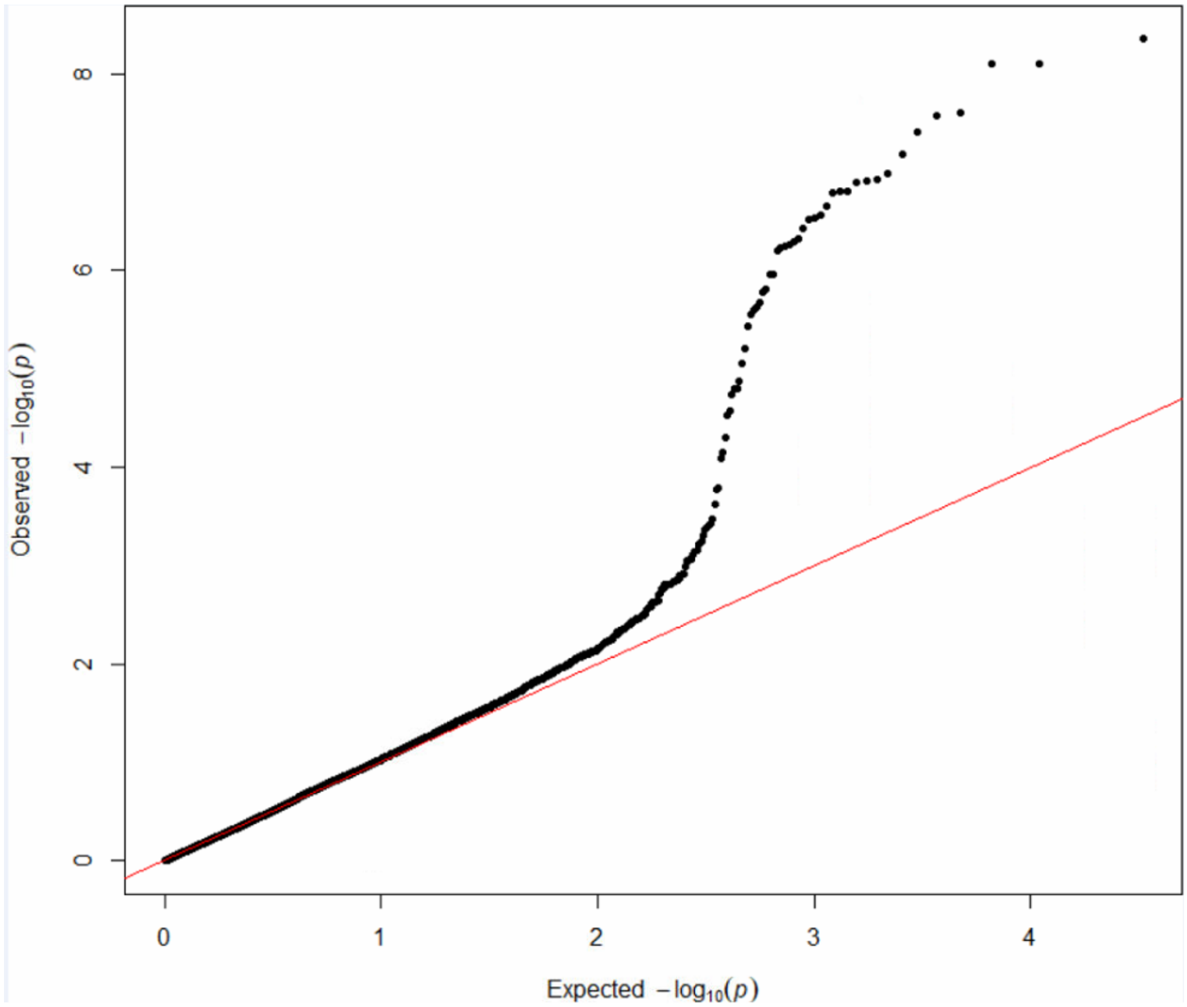


**Figure S1.** Quantile–quantile plot for Parkinson's disease.

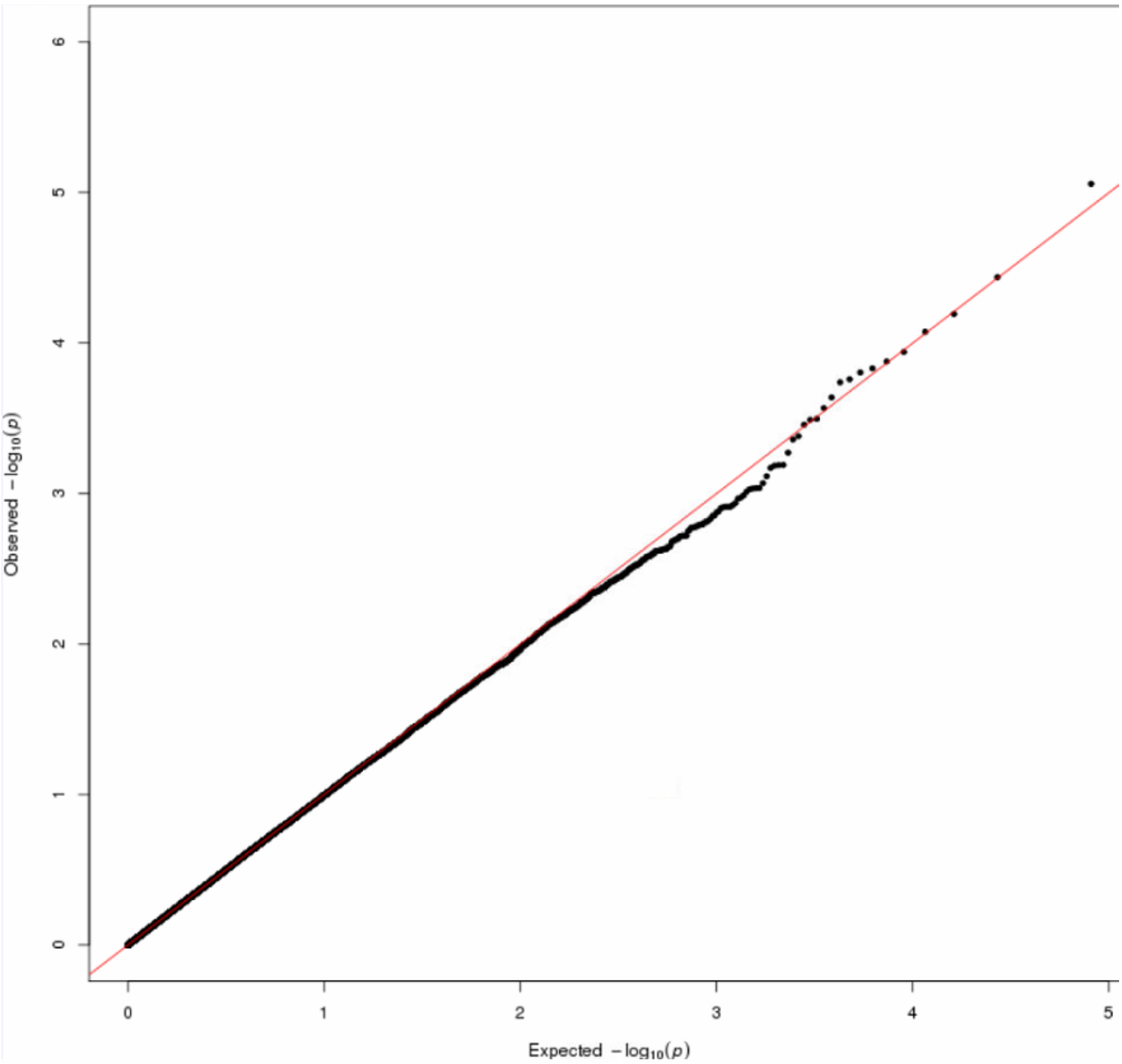


**Figure S2.** Quantile–quantile plot for dementia in Parkinson's disease.

**Table S1.** Characteristics of the markers used in the microarray.

| Source | Not Design | Design | Submit | Design Rate (%) |
|---|---|---|---|---|
| AD (PMID:30820047) | 689 | 244 | 933 | 26.2 |
| AD_IGAP | 9754 | 6474 | 16,228 | 39.9 |
| ADPD_cRE | 5412 | 533 | 5945 | 9.0 |
| DLB (PMID:31065058) | 2 | 1 | 3 | 33.3 |
| ENDOSOME | 451 | 714 | 1165 | 61.3 |
| GWAS_cat_AD | 2827 | 2319 | 5146 | 45.1 |
| GWAS_cat_DM | 484 | 332 | 816 | 40.7 |
| GWAS_cat_PD | 1356 | 1183 | 2539 | 46.6 |
| GWAS_cat_T2D | 6248 | 5261 | 11,509 | 45.7 |
| KEGG_LYSOSOME | 946 | 1289 | 2235 | 57.7 |
| KEGG_TOLL_LIKE_RECEPTOR_SIGNALING_PATHWAY | 817 | 1166 | 1983 | 58.8 |
| MITOCHONDRION | 2389 | 3458 | 5847 | 59.1 |
| NEURON_DEVELOPMENT | 423 | 715 | 1138 | 62.8 |
| PD (Nall_2019_Biorxiv) | 663 | 615 | 1278 | 48.1 |

| | | | | |
|---|---|---|---|---|
| PD (PMID:28892059) | 6 | 6 | 12 | 50.0 |
| PD_Foo | 3681 | 3604 | 7285 | 49.5 |
| PD_Nall | 13158 | 10,132 | 23,290 | 43.5 |
| REACTOME_ACTIVATION_OF_NF_KAPPAB_IN_B_CELLS | 378 | 545 | 923 | 59.0 |
| SLEEP | 142 | 282 | 424 | 66.5 |
| SLEEP (PMID:18820697) | 3 | 6 | 9 | 66.7 |
| SLEEP (PMID:19412176) | 5 | 5 | 10 | 50.0 |
| SLEEP (PMID:21170044) | 4 | 3 | 7 | 42.9 |
| SLEEP (PMID:22257907) | 7 | 4 | 11 | 36.4 |
| SLEEP (PMID:26507264) | 34 | 50 | 84 | 59.5 |
| SLEEP (PMID:29535854) | 139 | 107 | 246 | 43.5 |
| T2D (PMID:30718926) | 9 | 2 | 11 | 18.2 |
| T2D_Xue | 9223 | 8059 | 17,282 | 46.6 |
| UBIQUITIN_CYCLE | 262 | 426 | 688 | 61.9 |
| Other_GWAS_cat | 50,292 | 61,726 | 112,018 | 55.1 |
| Total | 109,804 | 109,261 | 219,065 | 49.9 |

**Table S2.** Staged verification of the markers.

| Step-by-step Marker Check for Tagging | | | |
|---|---|---|---|
| **Steps** | Description | Number of Markers | Number of QC Marker |
| Step0 | Raw | 219,065 | - |
| Step1 | Remove duple Marker | 179,344 | 39,721 |
| Step2 | 1000genome | 165,822 | 13,522 |
| Step3 | MAF = 0 | 151,641 | 14,181 |
| Step4 | Tagging $r^2$ 0.8 | 59,920 | 91,721 |
| Tagging SNP Coverage | | | |
| Step4 | Tagging r2 0.8 | 59,920 | 14,811 |
| Step5-1 | independent Marker | 45,109 | - |
| Step6-1 | Design Possible | 32,379 | 12,730 |
| Step5-2 | Non Independent Group | 14,807 | - |
| Step6-2 | Design Possible Group | 13,880 | 927 |
| Step7 | Final Tagging SNP | 46,259 | - |

**Table S3.** Additional selection according to selection priority.

| | Description | Number of Markers | Cumulative Numbers | Priority |
|---|---|---|---|---|
| Step0 | Tagging SNP | 46,259 | 46,259 | Top |
| Step1 | Priority1 Uniq Marker | 12,088 | 58,347 | Middle |
| Step2 | Priority2 Uniq Marker | 15,210 | 73,557 | Bottom |
| Step3 | Includes markers of interest and multi-allelic markers | 667 | 74,224 | Top |
