# Supplementary figures and images for: Microarray Genotyping Identifies New Loci Associated with Dementia in Parkinson’s Disease
Source: Genes (Basel). 2021 Dec 10;12(12):1975. doi: 10.3390/genes12121975 (PMC8701809; doi:10.3390/genes12121975)

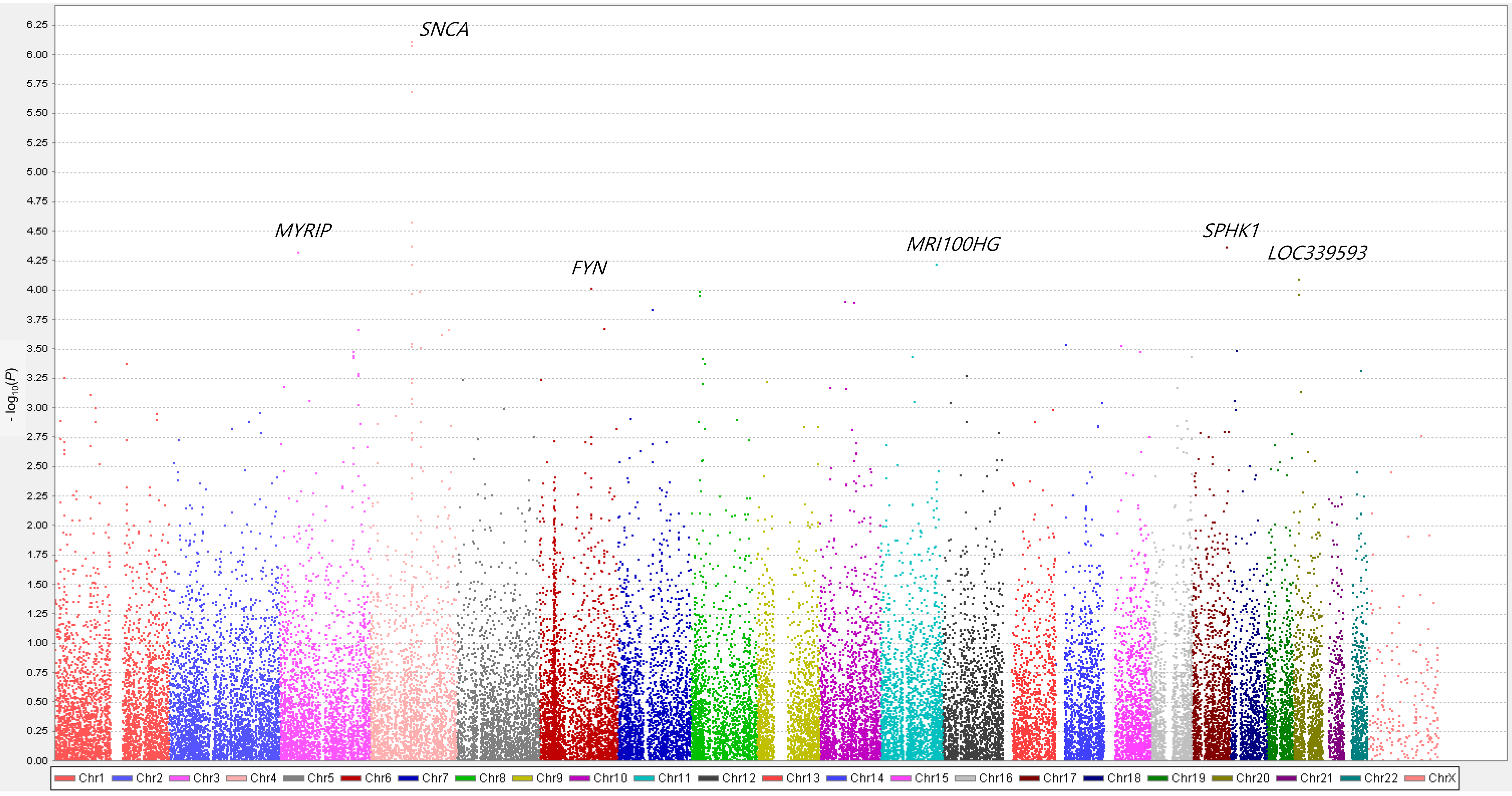

Supplement: Supplementary file 1 [file genes-12-01975-s001.zip › figure s1.tif]

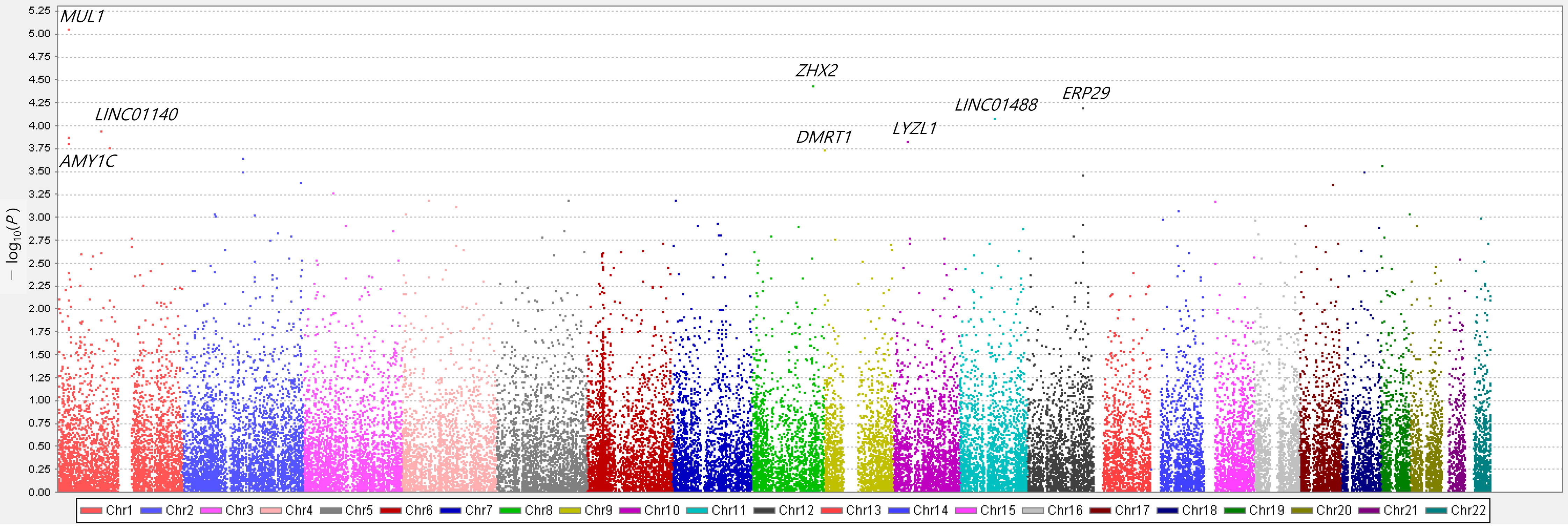

Supplement: Supplementary file 1 [file genes-12-01975-s001.zip › figure s2.tif]

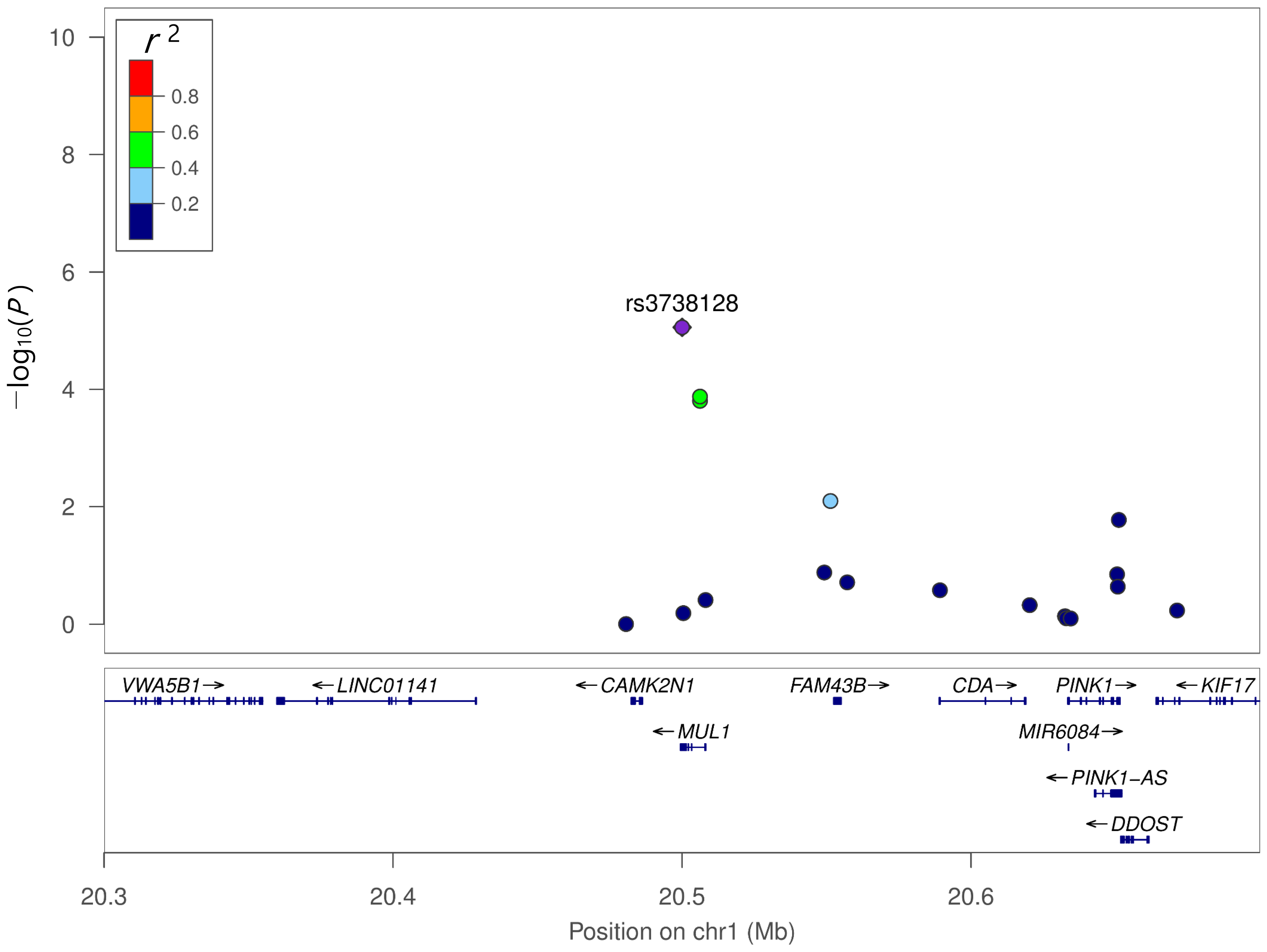

Supplement: Supplementary file 1 [file genes-12-01975-s001.zip › figure s3.tif]
